# Knowledge, practice, and challenges in the use of stainless steel crowns and the Hall technique among Yemeni general dental practitioners: a cross-sectional study

**DOI:** 10.3389/froh.2025.1527355

**Published:** 2025-06-20

**Authors:** Sarah Al-Rai, Mohanad Alyousefy, Zainab Al-Twaili, Eshraq Anqa, Mohammed Amin, Jameil Al-bateit, Halah Al-Mogahed, Ola B. Al-Batayneh

**Affiliations:** ^1^ Department of Conservative, Preventive Dentistry and Orthodontics, Faculty of Dentistry, Saba University, Sana’a, Yemen; ^2^ Chair of Epidemiology, School of Medicine and Health, Technical University of Munich, Munich, Germany; ^3^ Department of Oral Medicine and Periodontology, Faculty of Dentistry, Sana'a University, Sana’a, Yemen; ^4^ Department of Preventive Dentistry, Faculty of Dentistry, Jordan University of Science and Technology, Irbid, Jordan; ^5^ XP Dental Center, Sana'a, Yemen; ^6^ Department of Restorative and Esthetic Dentistry, Faculty of Dentistry, Sana'a University, Sana'a, Yemen; ^7^ Department of Orthodontics, Pediatric Dentistry and Community Dentistry, College of Dental Medicine, Sharjah University, Sharjah, United Arab Emirates

**Keywords:** stainless steel crown, Hall technique, general dental practitioner, questionnaire, Yemen

## Abstract

**Objectives:**

Stainless steel crowns (SSCs) and Hall technique (HT) are used to restore decayed primary molars. This study aimed to critically evaluate the practice, knowledge, and challenges faced by general dental practitioners (GDPs) in Yemen when restoring caries-affected primary molars using stainless steel crowns (SSCs) and the Hall technique (HT) in their routine clinical practice.

**Methods:**

A web-based cross-sectional survey was randomly distributed among GDPs registered with the Yemeni Dental Association using a Google Form (Google LLC, California, USA). The questionnaire comprised 21 questions organized into four sections: demographic information, SSC and HT practice, knowledge of SSC and HT, and challenges in SSC placement and HT use.

**Results:**

A total of 501 participants were included in the final analysis, with (54.7%) were females and (45.3%) were males. The majority graduated from public universities (68.5%). SSCs were used in daily practice by 51.3%, primarily for multisurface caries (67.3%). A low percentage used HT (30.1%), with the main concern being insertion difficulty due to lack of preparation (43.5%). Age and residency place significantly influenced SSC usage (*p* = 0.01 and *p* < 0.001, respectively). Frequent SSCs use was higher among dentists >30 years and those with >10 years of experience (*p* < 0.001). No demographic factors were associated with HT use. However, females and younger practitioners <30 were more likely to report needing additional practical training (*p* = 0.023 and *p* = 0.04, respectively).

**Conclusion:**

Enhancing GDPs' knowledge and skills in SSCs and HT through updated curricula and training could improve and enhance pediatric dental care.

## 1 Introduction

The prevalence of dental caries in children remains high worldwide affecting both primary and permanent dentition (1). According to the World Health Organization, 45% of children aged 1–9 in Yemen suffered from untreated tooth decay, reflecting the country's underdeveloped healthcare conditions (2). Selecting the most appropriate treatment option for primary teeth is one of the dentistry challenges. Caries management approaches differ according to the patient's caries risk level, which plays a key role in guiding the clinician's treatment decisions (3). Clinicians' decisions are influenced by several factors as patient's age, motivation, caries risk, tooth status, caries stage, and the dentist's expertise (4, 5).

Preformed metal crowns (PMCs) are one of the most common treatment options for restoring decayed and badly destroyed teeth (6). They were introduced into pediatric dentistry in 1947, described by Engel, and then disseminated by Humphrey in 1950 (7, 8). Stainless steel crowns (SSCs) have properties that make them widely used and selected in pediatric dentistry, as they give superior clinical performance with better longevity and low cost (6).

Hall Technique (HT) can be defined as the placement of PMCs without carious removal, tooth preparation, or local anesthesia. PMCs are cemented using glass-ionomer (luting) cement over carious primary molars (6). Since its inception, the technique has gained considerable attention due to its simplicity, effectiveness, and acceptability among both practitioners and patients. Among all carious management techniques, the overall success rate of HT was 49%. Moreover, its success rate was 80% in comparison with other restorations. On the other hand, HT had a similar success rate when compared to conventional PMCs (6). General dental practitioner (GDPs) have expressed positive views on the use of the Hall Technique and stainless steel crowns for restoring primary molars. The technique simplicity and effectiveness, along with the reduced need for advanced behavior management techniques, contribute to its favorable reception within the dental community (6).

In Yemen pediatric dentistry is underserved specialty, with only seven consultants and six experts in the capital city according to Yemen Dental Association. Many pediatric dentists trained abroad did not return due to the country's unstable situation. As a result, GDPs are under heavy demand to perform such treatments. SSCs have been used in Yemeni dental schools and clinics for 11 years. This study hypothesizes that GDPs exhibit variable levels of knowledge, attitudes, and practices regarding the use of SSCs and HT, potentially influenced by differences in educational background, clinical training, and professional experience. Given the established efficacy of SSCs and HT in pediatric dentistry, evaluating these factors among GDPs is essential to identify existing knowledge gaps and inform the development of targeted educational strategies to enhance clinical practice and patient outcomes.

## 2 Methods

### 2.1 Ethical approval

This web-based cross-sectional study protocol was obtained and approved by the Institutional Review Board and Institutional Ethics Committee of Saba University in compliance with Declaration of Helsinki and following the STROBE guidelines (approval no. 22/12).

### 2.2 Sample calculation

*A priori* sample size estimation was conducted to determine the minimum number of GDPs required for this cross-sectional study. The target population comprised 6,840 GDPs registered with the Yemen Dental Association. The calculation was performed using Cochran's formula for categorical data, assuming a 95% confidence level (*Z* = 1.96), a 5% margin of error (*e* = 0.05), and a response distribution (*p*) of 50%, which is standard when the true population proportion is unknown. This yielded a minimum required sample size of 384 participants. To enhance the study's robustness and account for potential non-responses, a total of 501 participants were included in the final analysis. A *post hoc* power analysis was performed using G*Power software (version 3.1), targeting a medium effect size (Cohen's *w* = 0.3), with a significance level set at *α* = 0.05 (two-tailed). The resulting statistical power was 99%, confirming the adequacy of the sample size for detecting meaningful associations using the Chi-square test.

### 2.3 Questionnaire development and validation

The survey was developed by the comprehensive review of existing literature on SSCs and HT. Most of questions were adapted from existing literature (Santamaria et al., 2018; 9, 10). Additional questions were developed after consultation of two experts (SA, OB) to check content validity of all questions and ensure that questions were relevant and comprehensive. After that a pilot study was conducted by distributing the questionnaire to 10 GDPs to assess clarity and reliability. The Pilot testing showed no significant mistakes regarding the questionnaire design; furthermore, participants showed no significant difficulties in answering questions. In general, the overall format of the questionnaire remained unchanged, and no questions were removed or added. Moreover, the results of the survey were not included in the final results.

### 2.4 Questionnaire distribution and data collection

This questionnaire was created using Google Form (Google LLC, California, USA) and distributed for each GDP via WhatsApp (Meta Platforms Inc., California, USA) between January and April 2023. The survey included an introductory section summarizing the study aims and obtaining informed consent prior to start the questionnaire. Participation was voluntary and anonymous, with no personal or identifying information collected. The questionnaire was composed of 21 questions and divided into four parts: The first part (Q1-5) was demographic data and general information about the GDP, the second part (Q6-15) was about SSC and HT practice, and the third part (Q16-19) was about knowledge regarding SSC and HT and the last part (Q20-21) was about challenges during SSC placement and HT use.

### 2.5 Data analysis

Data was analyzed using SPSS version 28 (Inc., Chicago, IL) USA). The categorical variables were presented by frequencies and percentages. The chi-square or Fischer exact test was used to assess the association between the categorical variables such as age, gender, region, experience, and SSC/HT use. Odds ratios (OR) and 95% confidence intervals (CI) were calculated to quantify the strength of these associations. A *p*-value of <0.05 was considered statistically significant.

## 3 Results

### 3.1 Demographic data

After excluding 30 respondents practicing outside Yemen, 501 participants were included in the final analysis. Females were (54.7%), and most were under 30 year-olds (61.5%). Most participants resided in the northern region (62.9%), with 23.4% in the central region. In terms of education, 68.5% graduated from public universities, and 62.5% had less than 10 years of professional experience (Table 1).

**Table 1:** Demographic data of the questionnaire respondents.

Demographic characteristics	Respondents *N*	%
1. Gender		
Males	227	45.3
Females	274	54.7
2. Age		
<30	308	61.5
>30	193	38.5
3. Residency area		
The North area	315	62.9
The middle area	117	23.4
The south area	69	13.8
4. Which university did you graduate from?		
Public	343	68.5
Private	158	31.5
5. Years of experience		
<10	313	62.5
>10	188	37.5

Data are presented as numbers (%).

### 3.2 Practice, knowledge and challenges face GDPs during SSCs and HT application

Regarding practice part questions, data illustrated that most participants (95%) treated pediatric patients in their clinics; moreover, 60.1% of the participants treated less than 10 children per week. Most of these children ages (56.9%) were over 6 year-olds. Approximately half of the questionnaire respondents (51.3%) incorporated SSCs into their daily practice. From the previous percentage, only 19% used SSCs more frequently over seven times per week; furthermore, 64.3% of respondents performed SSC less than five times during their undergraduate training. Regarding HT, 66.7% of the respondents reported that they did not use it, while 3.2% admitted that they were unfamiliar with the technique. The majority of the sample (82.6%) agreed that they needed extra training on SSCs and HT because most of the responses (65.3%) agreed that they had deficiency in their theoretical and practical training in their universities. Moreover, video demonstration (51.3%), hands-on courses (44.1%), and lectures (44.3%) were the most common ways selected for gaining training.

Regarding knowledge about SSCs and HT, 77% of the respondents learned SSC in the university, while about half of the participants (50.7%) learned HT in their university education. On the other hand, 26.5% of the GDPs acquired knowledge through YouTube, and only 8.4% attended workshops or conferences. Some other respondents (13.2%) mentioned that they learned about HT through social media such as Facebook and Instagram and attended some online lectures and webinars through Zoom and other online programs. GDPs agreed that the primary reasons for using SSCs were multisurface caries (67.3%) and after pulp therapy (60.5%), whereas infra-occluded teeth were the least common indication (12.4%). According to the respondents, the advantages of SSCs use included maintaining function and occlusion (79.6%), long durability (57.3%), good prognosis (48.7%), and the availability of scientifically documented treatments (21.6%).

Challenges faced GDPs during SSC placement were mainly patient cooperation (44.9%) and economic issues (35.7%). In contrast, a smaller percentage cited their preference for esthetic materials (17%) as a reason for not using SSCs. Some other respondents mentioned other causes, such as the unavailability of the material in their clinics or parents' compliance. However, the primary concern regarding HT was the difficulty in insertion of SSCs due to lack of preparation (43.5%), followed by concerns about caries removal (43.3%). The least concern reported was pain, as there was no need for giving anesthesia (17.4%) (Table 2).

**Table 2:** Practice, knowledge and challenges faced by GDPs during SSCs and HT application.

Question	Respondents *N*	Percentage (%)
6. Do you treat children in your clinic?		
Yes	476	95
No	25	5
7. Number of children you treat per week		
None	40	8
<10	301	60.1
>10	160	31.9
8. Age of child you treat per week		
None	23	4.6
<6	193	38.5
>6	285	56.9
9. Do you use SSC in your daily practice?		
Yes	257	51.3
No	244	48.7
10. How many SSCs do you use per week?		
None	196	39.1
<7	210	41.9
>7	95	19
11. How many SSCs have you done during your undergraduate training?		
<5	322	64.3
>5	179	35.7
12. Do you use the Hall technique?		
Yes	151	30.1
No	334	66.7
I do not know the technique	16	3.2
13. Did you get enough theoretical and practical instructions regarding SSC and Hall technique during your undergraduate study?		
Yes	174	34.7
No	327	65.3
14. Do you need more practice regarding stainless steel crown and Hall technique?		
Yes	414	82.6
No	87	17.4
15. If yes, how do you want to get more information and practice?		
Lectures	222	44.3
Illustrated guidelines	76	15.2
Hand-on course	221	44.1
Video demonstration	257	51.3
Webinar	68	13.6
16. How did you learn about SSC placement?		
In university	386	77
Workshop/conferences	23	4.6
Articles	16	3.2
Youtube	61	12.2
Others/mention	15	3
17. How did you learn about the Hall technique?		
University	254	50.7
Workshop/conferences	42	8.4
Articles	69	13.8
YouTube	133	26.5
Others/ mention	66	13.2
18. Most indications for using/placing SSC is/are:		
Multisurface caries	337	67.3
After pulp therapy	303	60.5
Developmental anomalies	91	18.2
High caries risk patients	177	35.3
Fractured teeth	193	38.5
Child with bruxism	73	14.6
Infraoccluded teeth	62	12.4
19. Advantages of using SSC		
Maintain function and occlusion	399	79.6
Keeps tooth symptomless	130	25.9
Scientifically documented treatment	108	21.6
Long durability	287	57.3
Good prognosis	244	48.7
20. Causes of avoiding the use of SSC		
Technique complexity	95	19
Aesthetic concern	122	24.4
Patient cooperation	225	44.9
Time-consuming	91	18.2
Low caries prevalence	105	21
Lack of knowledge or training	100	20
Preferring esthetic material	85	17
Economic reason	179	35.7
Others/mention	31	6.2
21. What is your primary concern regarding the Hall technique?		
No caries removal	217	43.3
Pain because there is no anesthesia	87	17.4
Difficulty in insertion because there is no preparation	214	43.5
Increase vertical dimension after crown cementation	149	29.7

Data are presented as numbers (%).

### 3.3 The association between demographic characteristics and the use of SSCs

**Figure 1:**
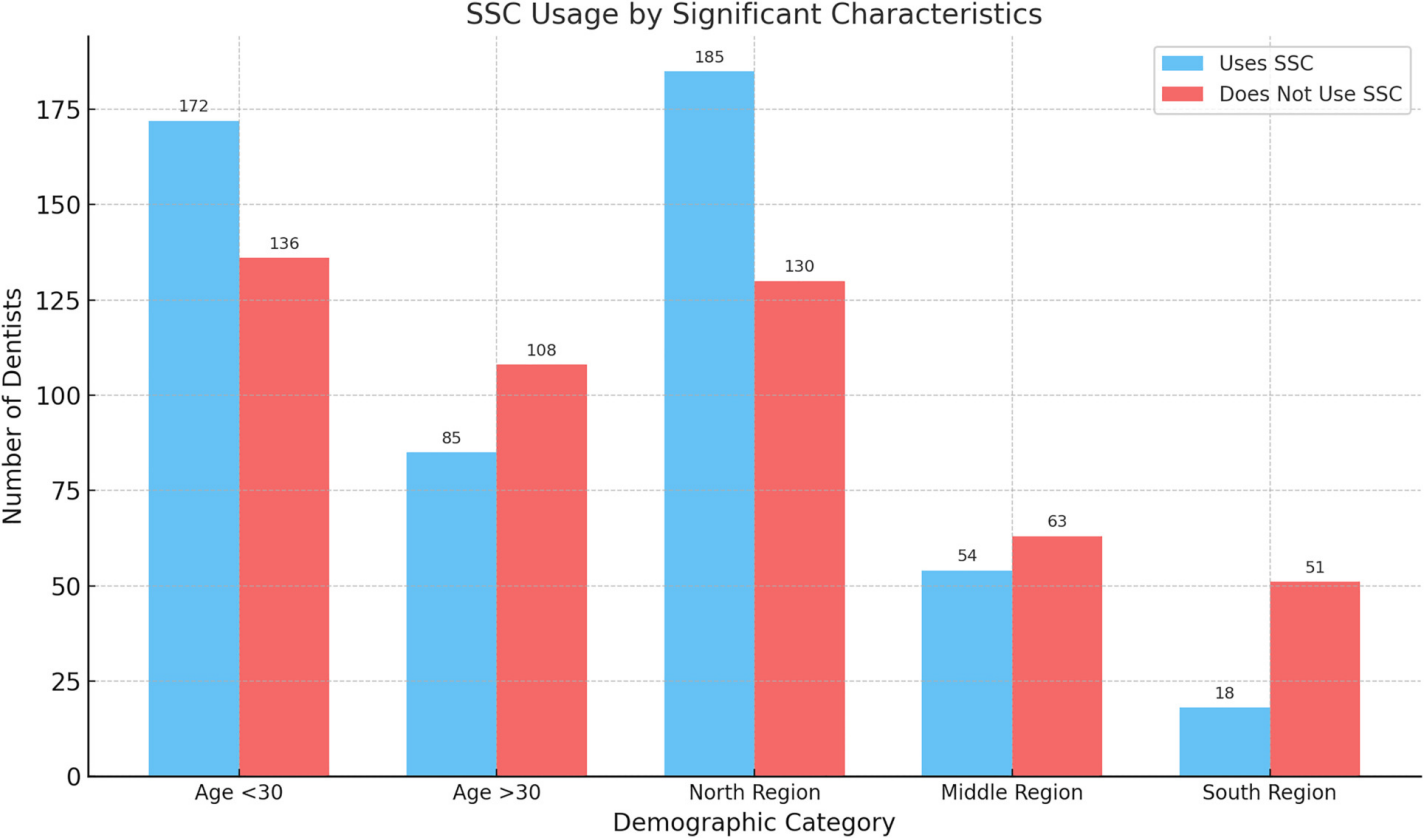
Distribution of SSC usage among GDPs by significant factors: age and region.

**Figure 2:**
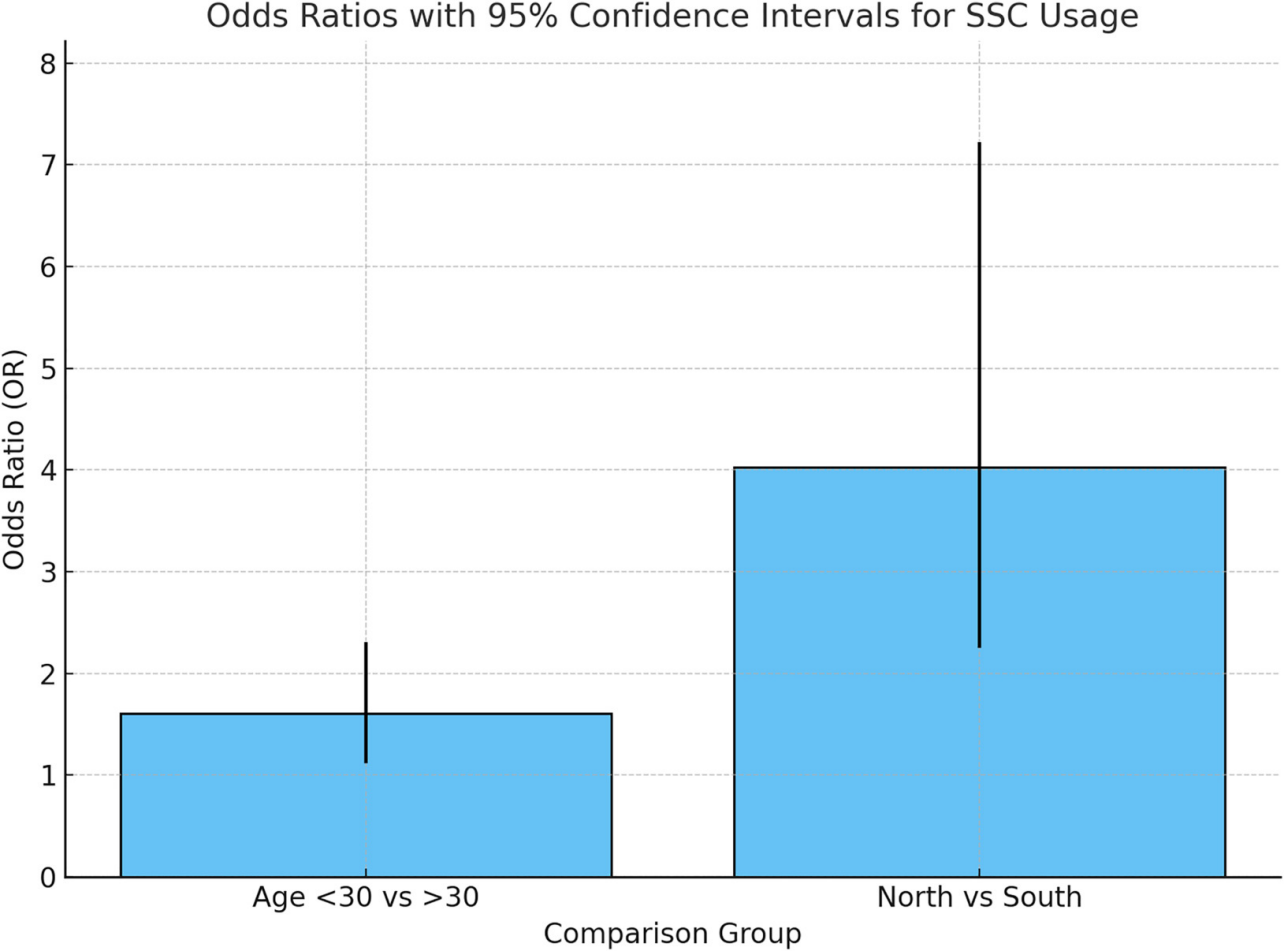
Impact of age and region on SSC usage.

Results showed that there were two factors that were significantly played role in the use of SSCs: age and geographic residency. Age was found to have a significant role in SSCs use; dentists under 30 year-olds used SSCs more than dentists over 30 years old (OR = 1.61; 95% CI: 1.12–2.31; *p* = 0.01). Geographical location is associated with the use of SSCs; GDPs who were practicing in the north used SSCs more the GDPs in other areas (OR = 4.03, 95% CI: 2.25–7.22; *p* < 0.001) (Figures 1, 2).

### 3.4 The association between demographic characteristics and the frequency of SSCs use per week

**Figure 3:**
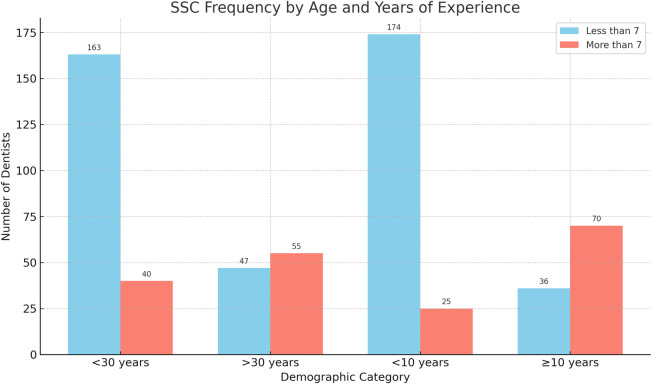
Distribution of frequency of SSC usage per week among GDPs by significant factors: age and years of experience.

**Figure 4:**
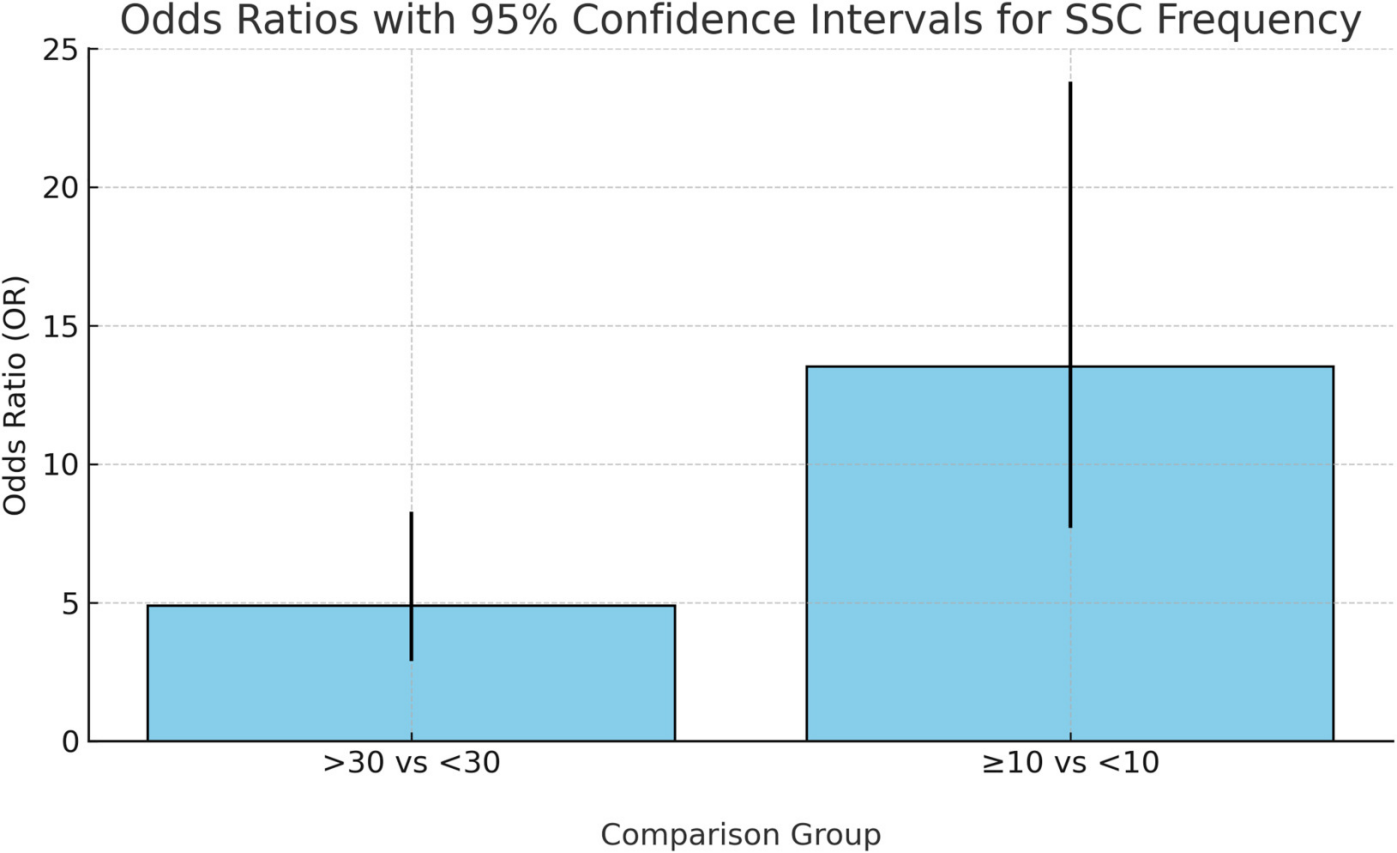
Impact of age and years of experience on frequency of SSC usage per week.

The rate of SSCs use per week was affected significantly by two factors: age and years of experience. Dentists over 30 year-olds were significantly more likely to use SSCs more than seven times per week compared to their younger counterparts under 30 year-olds (OR: 4.89, 95% CI: 2.89–8.27; *p* < 0.001). Similarly, dentists who had >10 years of experience demonstrated a markedly higher likelihood of frequent SSCs use compared to those <10 years of experience, (OR: 13.53, 95% CI: 7.69–23.81; *p* < 0.001) (Figures 3, 4).

### 3.5 The association between demographic characteristics and the use of the HT

This study revealed that there was no association between the demographic data and the use of HT.

### 3.6 The association between demographic characteristics and the need for more practice regarding SSCs and HT

**Figure 5:**
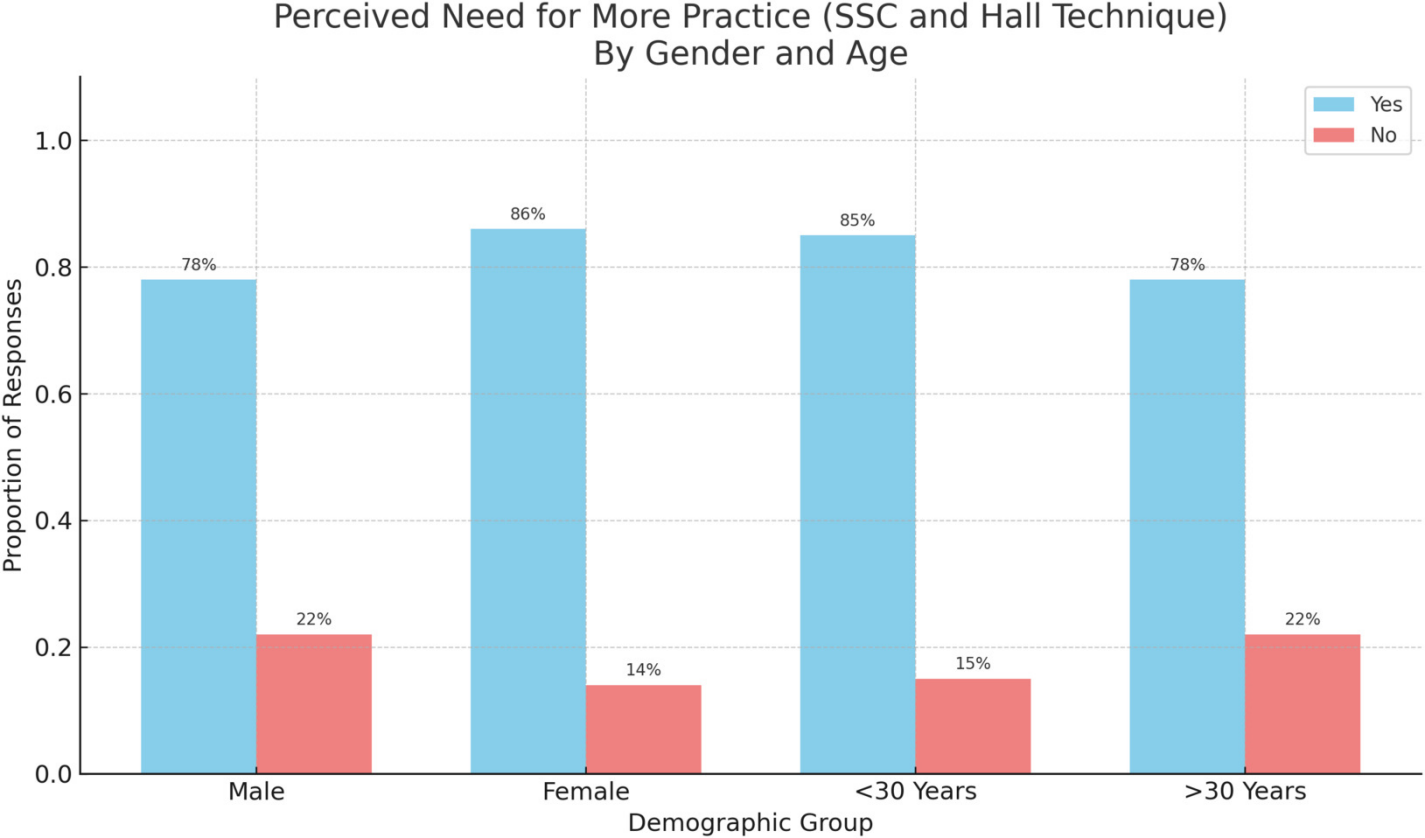
Gender and age-based differences in perceived need for further SSC and HT training.

**Figure 6:**
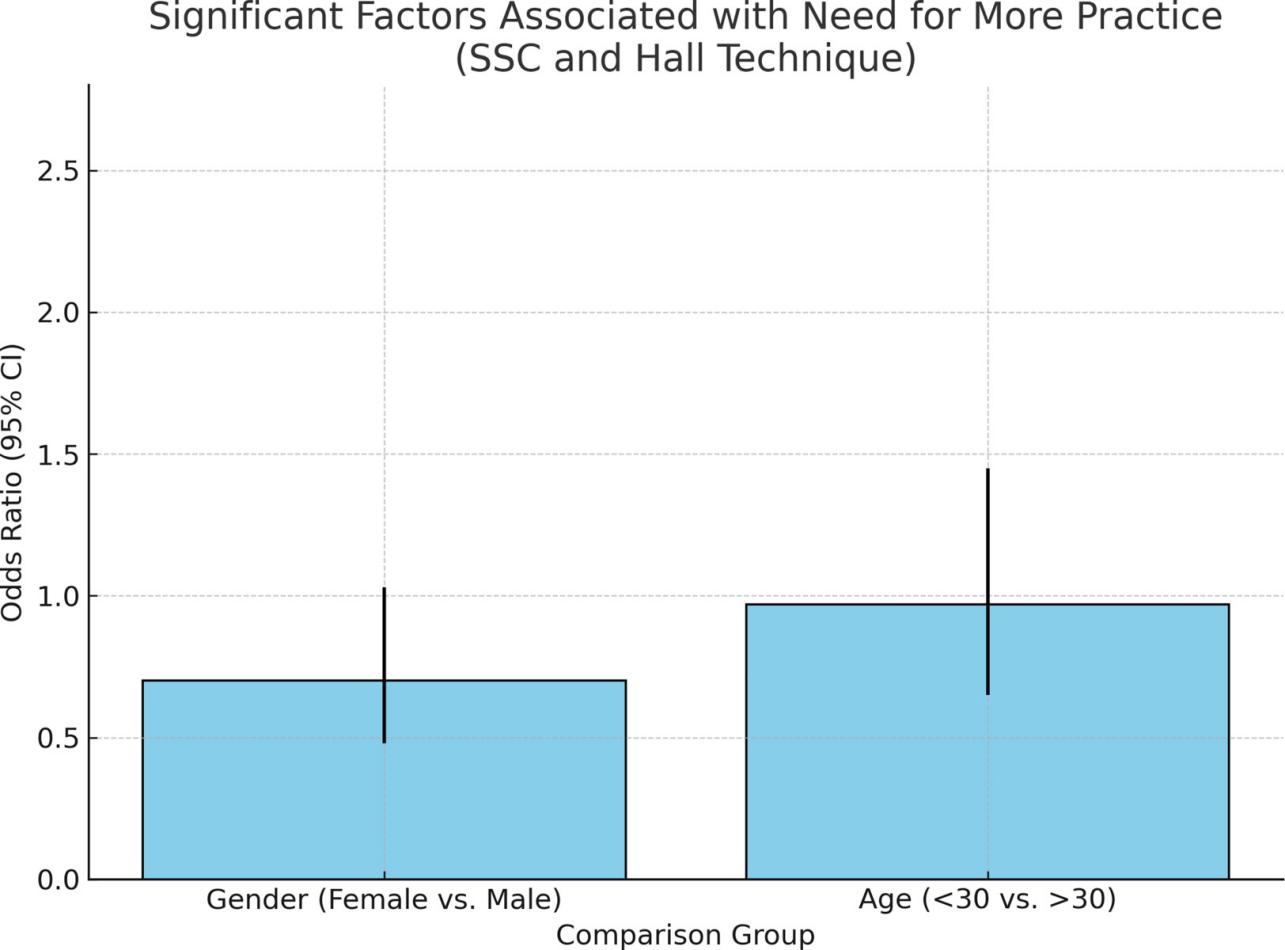
Impact of gender and age on the perceived need for further SSC and HT training.

The results of this study showed two significant factors associated with need for more practice regarding SSCs and HT: gender and age. A higher percentage of females compared to males responded that they need more practice regarding SSCs placement and HT (OR: 1.71, 95% CI: 1.07–2.73; *p* = 0.023). Moreover, Respondents under the age of 30 year-olds showed that they need more practice regarding SSCs and HT more than respondents over 30 year-olds (OR: 1.62, 95% CI: 1.02, 2.59; *p* = 0.04) (Figures 5, 6).

### 3.7 The association between demographic characteristics and whether they get enough theoretical and practical instructions during undergraduate study regarding SSCs and HT

**Figure 7:**
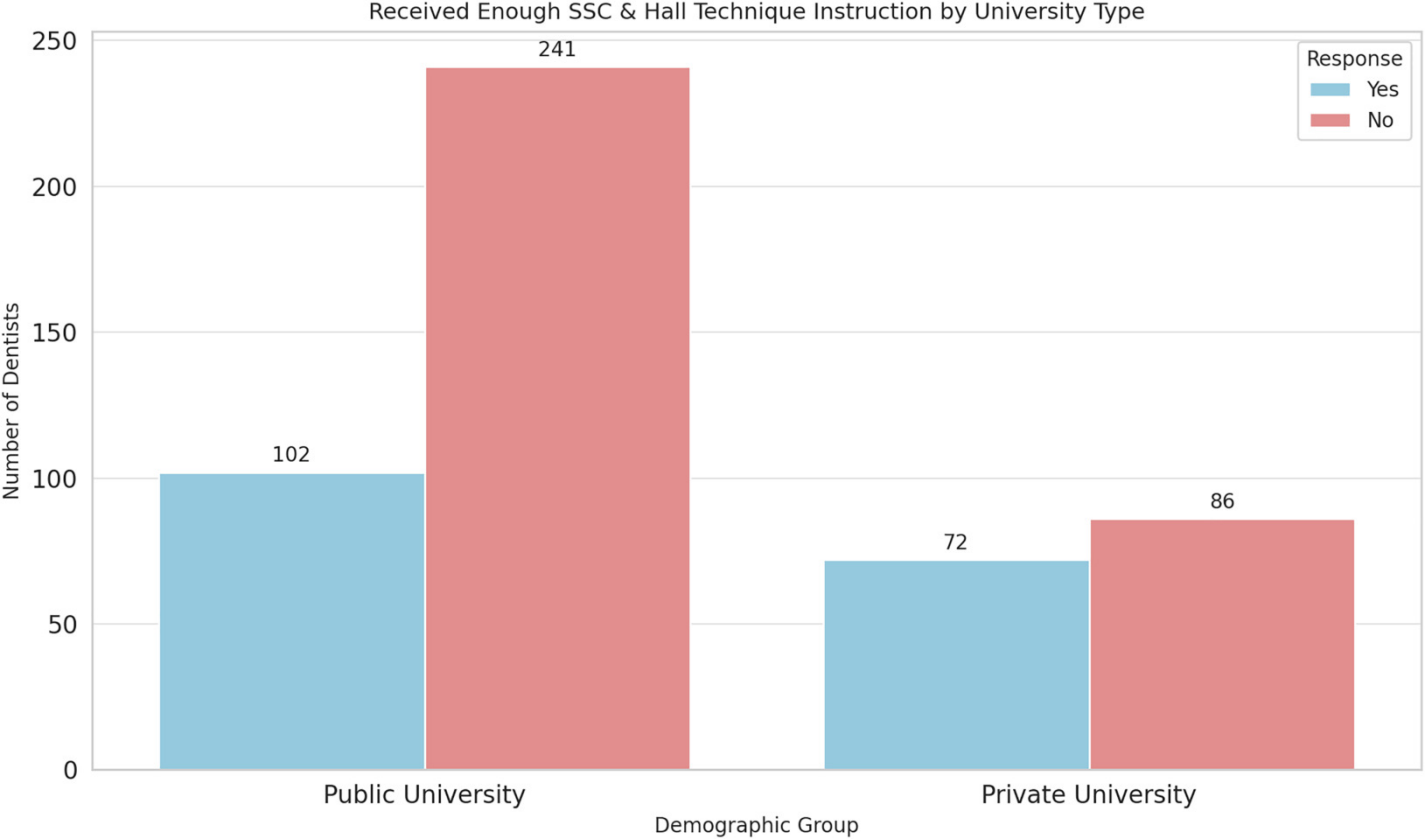
Efficiency of SSC and HT training based on educational type.

**Figure 8:**
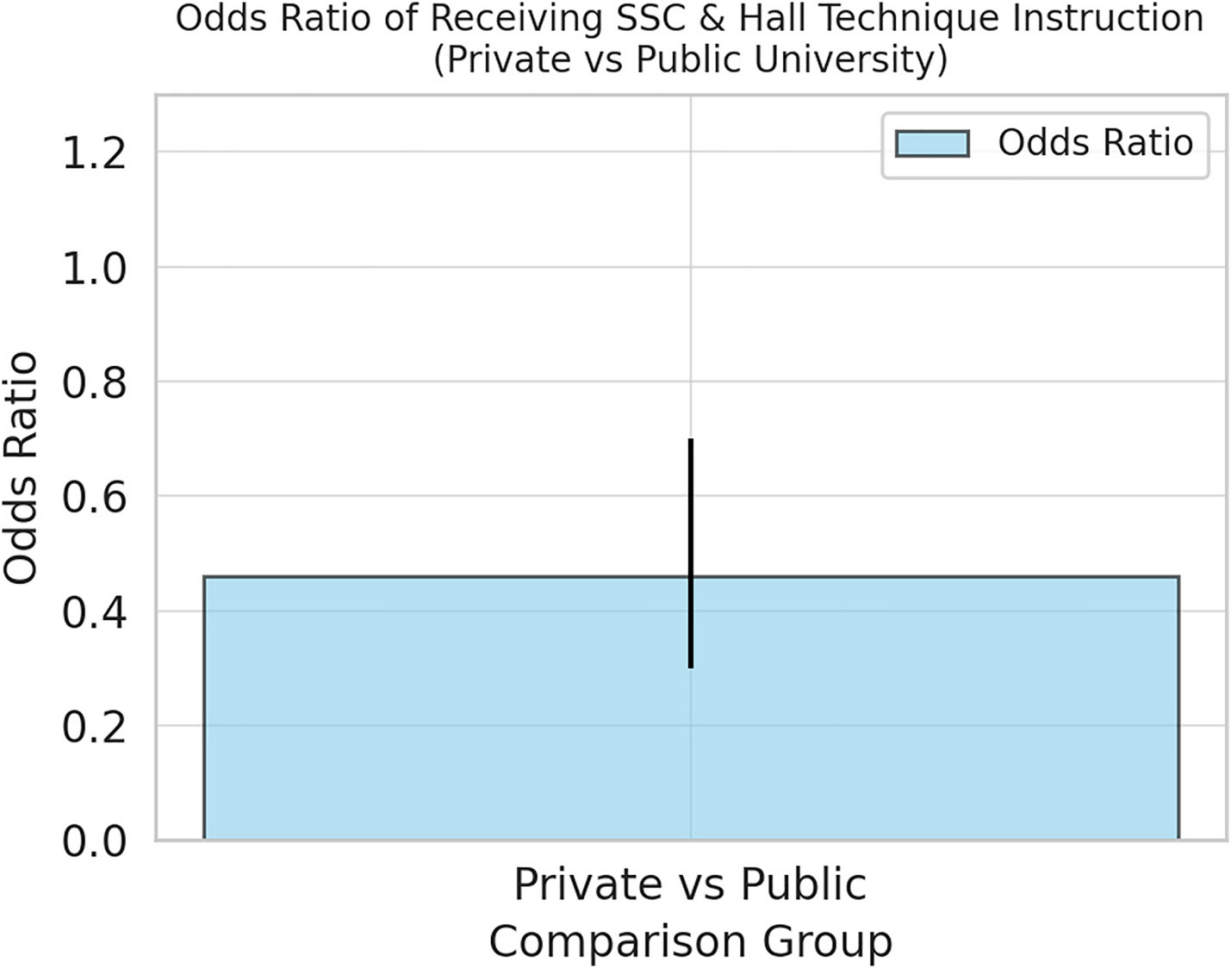
Impact of educational type on SSC and HT training.

The university of graduation (public or private) was the only factor that can affect whether the respondents received enough theoretical and practical instructions during their undergraduate study regarding SSCs and HT. In another words, GDPs who graduated from private universities received more training than GDPs graduated from public universities (OR: 0.51, 95% CI: 0.34–0.75; *p* ≦ 0.001) (Figures 7, 8).

## 4 Discussion

This study aimed to assess practice, knowledge and challenges of GDPs in Yemen regarding the use of SSCs and HT among dentists in Yemen, as well as their demographic features and training requirements. To the best of our knowledge, this is the first study investigating the usage of both SSCs and HT among Yemeni GDPs.

Interestingly, most of the respondents were female (54.7%), showing a significant gender imbalance in Yemen's dental profession. This result suggests that dental profession in Yemen may have a higher proportion of females compared to males. This observation is consistent with comparable patterns reported in other studies. For instance, a survey conducted among Norwegian and Finnish dentists indicated a higher proportion of females than males, indicating the persistent gender imbalance within the dental sector (9). Furthermore, 61.5% of participants were under 30 year-olds, indicating a young and potentially dynamic workforce. Geographically, most respondents (62.9%) resided in the North area, which correlates with concentrated dental infrastructure in Northern Yemen, more specifically in Sana'a. This may explain the relatively higher use of SSCs and HT in this region, given better access to materials and clinical facilities (11). In contrast, the southern regions continue to be affected by active conflict zones, which have significantly limited access to crown materials and dental laboratories, potentially restricting the use of these treatment options (12). Moreover, a significant number of dentists (68.5%) graduated from public universities demonstrating the dominance of public institutions in dental education. Yemen's reliance on public dental education aligns with low- and middle-income countries (LMICs) like Egypt and Pakistan, where 70%–80% of dentists were trained in public schools due to centralized healthcare policies (13, 14).

Our findings indicated that while most surveyed dentists in Yemen treated pediatric patients, only 51.3% of respondents reported using SSCs in their daily practice. This rate exceeded the 26.3% SSCs adoption reported by Al-Arwali et al., which was based exclusively on data from Sana'a only (11). In contrast, our study encompassed northern, middle and southern of Yemen, thereby reflecting a wider spectrum of dental practice environments and regional differences in the utilization of SSCs. Comparatively, a study in Germany found that 66% of the respondents did not use SSCs (4). Similarly, in India a study by Bedre and Gurunathan showed only 46.4% of participants used SSCs in their daily practice (15). However, the context in Yemen is distinct.

The limited use of SSCs may be shaped by several local factors, including insufficient training in pediatric restorative techniques, economic constraints, and the lack of clear national guidelines or policies supporting the routine use of SSCs. Furthermore, ongoing conflict and regional disparities in dental infrastructure, especially between the more equipped northern regions and the conflict-affected south, may further hinder accessibility to crown materials and limit clinical capacity. These variables likely play significant roles in shaping dentists' decisions regarding the incorporation of SSCs into their clinical practice. Addressing these factors could help in bridging the gap and promote more consistent adoption of SSCs, which are beneficial for pediatric dental care.

Our findings revealed no significant association between gender and SSCs use (*p* = 0.6), aligning with a 2024 study from Yemen which similarly reported no gender-based difference in SSCs adoption (*p* = 0.201) (11).

In contrast, age demonstrated a significant influence (*p* = 0.01), Younger dentists, particularly those under 30s, were more likely to utilize SSCs than their older counterparts. This finding may suggest that younger dentists were more receptive to adopting knowledge and training in many fields like SSCs and HT, potentially due to recent advancements in dental education or evolving treatment protocols. Moreover, SSCs use was more common in the northern and middle areas compared to the southern region (*p* < 0.001). This regional variation in SSCs utilization may reflect differences in patient demographics, socioeconomic factors, or the availability of dental healthcare resources.

Notably, years of experience also influenced the frequency of SSCs use, with less experienced dentists using SSCs more frequently (*p* < 0.001). This finding could be explained by the fact that newer graduates may have received more recent training on SSCs placement and may be more inclined to incorporate them into their practice. A study showed that dentists with ≤5 years of experience have been shown to prescribe SSCs 2.5 times more often than those with over 15 years of experience (*p* <  0.001) (16). Furthermore, those who completed programs that included SSC-focused clinical rotations reported using them three times more frequently, underscored the impact of targeted training on clinical practice (17).

It was notable that a substantial portion of respondents learned about HT through informal channels such as YouTube (26.5%), suggesting a potential gap in formal educational curricula. This finding was consistent with a study conducted in China, where dentists typically learned about this technique through continuing education training courses and conferences (18). Contrast to this with the UK study, where 96% HT adoption followed undergraduate curriculum integration (19). Specifically, a survey of UK dental therapy schools found that the majority taught both conventional and alternative caries management techniques, including HT within their undergraduate programs (20). In Yemen, however, dental schools generally lack structured HT modules, leaving many practitioners to depend on peer learning or non-verified online resources, such as YouTube, to acquire the technique.

The expressed need for additional training on SSCs and HT (82.6% of the total sample) underscores a potential gap in current dental education programs in Yemen. Dentists preferred practical learning methods such as video demonstrations (51.3%) and hands-on courses (44.1%), suggesting a preference for experiential learning over theoretical instruction.

The significant impact of the university of graduation on the adequacy of theoretical and practical instructions regarding SSCs and HT (*p* ≦ 0.001) highlights potential discrepancies in dental education quality between public and private institutions. Specifically, graduates from private universities received more comprehensive instruction than public university graduates. This finding underscores the importance of curriculum standardization and continuous professional development programs to ensure that all dentists receive comprehensive training in evidence-based techniques.

Despite the high awareness of SSCs benefits, concerns such as patient cooperation (44.9%) and economic factors (35.7%) appear to influence their utilization. In a study by Uhlen et al., the primary barrier frequently reported for not using SSCs was a lack of practical training (9).

Findings related to HT utilization reveal noteworthy insights into the current practices and attitudes among dentists surveyed. A considerable proportion of respondents (66.7%) reported not using the HT, while a small percentage (3.2%) admitted to being unfamiliar with the technique altogether. This corresponded with findings from other studies conducted in China (18) and the USA (10), where the use of the HT was reported at 40.2% and 39%, respectively. This disparity in HT utilization across different countries could be attributed to variations in introducing and integrating the technique into dental education curricula. Notably, in the UK, where HT usage appeared to be higher (96%), it is likely because the technique was introduced into the undergraduate pediatric dentistry curriculum before 2010 (21). When comparing Yemen to other Arab contexts, such as Qatar, where 58% of pediatric dentists reported using HT, the difference becomes notable. This higher adoption in Qatar likely reflects the presence of a well-structured primary oral healthcare system, supported by stable infrastructure, continuous training, and clear clinical guidelines (22).

Gender and age were found not significantly influence HT use among dentists in Yemen. A lower percentage of males (33%) and females (27%) reported using HT. This aligns with a study conducted by Ding et al. reported that the use of HT showed no association with gender (18). In contrast to Gonzalez et al.'s findings, which showed a significant association between gender and HT use (10). This suggested potential regional differences in dental practices or training programs. Further investigation is necessary to elucidate these discrepancies comprehensively.

One of the primary concerns expressed regarding the HT was the difficulty in insertion due to a lack of preparation (43.5%). Multiple studies have reported that practitioners, especially those new to the HT, find crown insertion challenging because no tooth reduction is performed (23). In a clinical trial by Innes et al., 15% of crowns were deemed “incompletely seated,” largely because of these insertion difficulties, and tight contacts often necessitated the use of orthodontic separators to facilitate crown placement (24).

This finding highlights the importance of ensuring dentists receives adequate training and support to effectively implement HT in their clinical practice. Other studies, including those by Gonzalez et al. (10) and Ding et al. (18), have highlighted further concerns regarding HT. Specifically, dentists who had not utilized the HT expressed apprehensions regarding potential complications such as pulp inflammation or necrosis following its application (10, 18). These disparities may stem from differences in education systems, clinical guidelines, and cultural factors. Addressing these challenges requires enhanced training and ongoing professional development opportunities for dentists.

Despite these challenges, it is encouraging to note that pain, typically associated with traditional anesthesia, was not a significant concern among respondents utilizing HT (17.4%) in our study. This can be attributed to the minimally invasive nature of HT, which eliminates the need for local anesthesia, caries excavation, and tooth preparation (23). These characteristics reduce procedural discomfort and psychological distress, particularly in pediatric patients, thereby supporting HT suitability as a child-friendly, atraumatic restorative approach.

Lastly, our study observed that most of those who employed HT (30.1%) gained knowledge of the technique during their university education, emphasizing the importance of formal training for undergraduates. However, it is noteworthy that most dental students in Germany have yet to learn about nor use the HT (4), indicating potential variations in educational practices. This suggests a need to incorporate training on HT into dental education curricula to ensure that dentists are adequately equipped with the necessary skills to utilize this approach effectively in clinical practice.

## 4 Conclusion

This study revealed a significant gaps related to practice and knowledge of SSCs use and HT among GDPs in Yemen. This could be attributed to training deficiencies, cultural perceptions, and resource limitations. To address these challenges and improve pediatric dental care, it is recommended to implement comprehensive training programs for dental professionals, integrating SSCs and HT into educational curricula, establishing ongoing education initiatives, launching targeted awareness campaigns, and ensuring effective resource management. Not only this Yemen Dental Association should develop a policy regarding SSCs and HT. These steps are essential for enhancing the adoption of SSCs and HT, thereby advancing oral health care for Yemeni children.

## 5 Limitation

While the research offers insights into how Yemeni GDPs use SSCs and HT, it is essential to acknowledge some limitations;

1. Sampling Bias: The data collection from dentists registered with the Yemen Dental Association does not represent the entire dental practitioner population of Yemen. The study results cannot be considered representative for all dental practitioners operating in Yemen.
2. Self-Reported Data: The collected information relied on self-reported responses from dentists, which could be influenced by recall and social desirability biases. The participants might have chosen answers they believed were most acceptable rather than providing an accurate picture of their actual clinical work.
3. Cross-Sectional Design: The study's cross-sectional design makes establishing relationships between characteristics and using SSCs and HT challenging. Longitudinal studies or randomized controlled trials would offer evidence of these associations.
4. Lack of Response Rate Information: The research study failed to disclose the response rate along with information about non-respondents. The absence of non-respondent information creates the risk of non-response bias because non-respondents might differ substantially from participants in terms of characteristics and practices.
5. Generalizability: The research centered on dentists in Yemen. It is important to note that the results may not necessarily apply to regions with distinct healthcare systems, dental education structures, and cultural backgrounds. Therefore, it is crucial to be mindful when extending these findings to different populations.

Considering these constraints, future research efforts will enhance our knowledge of how dentists utilize SSCs and HT and support the creation of tailored interventions to improve care.

## Data Availability

The original contributions presented in the study are included in the article/Supplementary Material, further inquiries can be directed to the corresponding author.
